# Phylogeography of *Potamon ibericum* (Brachyura: Potamidae) identifies Quaternary glacial refugia within the Caucasus biodiversity hot spot

**DOI:** 10.1002/ece3.5078

**Published:** 2019-03-26

**Authors:** Elaheh Parvizi, Alireza Keikhosravi, Reza Naderloo, Samaneh Solhjouy‐Fard, Farahnaz Sheibak, Christoph D. Schubart

**Affiliations:** ^1^ School of Biology, College of Science University of Tehran Tehran Iran; ^2^ Department of Biology Hakim Sabzevari University Sabzevar Iran; ^3^ Department of Plant Protection, College of Agriculture and Natural Resources University of Tehran Karaj Iran; ^4^ Zoology and Evolutionary Biology University of Regensburg Regensburg Germany; ^5^ Present address: Department of Zoology University of Otago Dunedin New Zealand

**Keywords:** biodiversity hot spot, freshwater crabs, Georgia, mtDNA, species distribution modeling

## Abstract

Refugia are critical for the maintenance of biodiversity during the periods of Quaternary climatic oscillations. The long‐term persistence of refugial populations in a large continuous refugium has resulted in a homogenous pattern of genetic structure among populations, while highly structured evolutionary lineages characterize the restriction of refugial populations to smaller subrefugia. These mechanisms have resulted in the identification of hot spots of biodiversity within putative glacial refugia. We studied phylogeography of *Potamon ibericum* (Brachyura: Potamidae) in the drainages of the western Caucasus biodiversity hot spot (i.e., Colchis and the Caucasus) to infer spatial genetic structure and potential refugia for a freshwater crab in this region. These areas have traditionally considered as a refugium due to the presence of Tertiary relict species. We integrated population genetic data and historical demographic analysis from cytochrome oxidase subunit I sequences and paleoclimatic data from species distribution modeling (SDM). The results revealed the lack of phylogeographic structure and provided evidence for demographic expansion. The SDM presented a rather homogenous and large refugium that extended from northeast Turkey to Colchis during the last glacial period. In contrast to these findings, previous phylogeographic study on *P. ibericum* of the eastern Caucasus biodiversity hot spot (i.e., Hyrcania) identified multiple independent refugia. By combining these results, we explain the significance of this important western Palearctic hot spot of biological diversity in shaping the geographic distribution of intraspecific genetic diversity in a freshwater taxon.

## 1 INTRODUCTION

Climatic oscillations during the glacial‐interglacial cycles of the Pleistocene period had a significant influence on the present‐day patterns of biological diversity (Hewitt, 2000,2004). The most noticeable changes in the distribution pattern and population size of many species occurred during the last glacial maximum (LGM; 23–18 ka BP; Hewitt, 2004; Provan & Bennett, 2008) when the temperate‐adapted species of the Northern Hemisphere endured the adverse climatic conditions in southern glacial refugia (Stewart, Lister, Barnes, & Dalén, 2010). In the western Palearctic realm, the southern European refugia and their postglacial recolonization routes are quite well understood (reviewed in Taberlet, Fumagalli, Wust‐Saucy, & Cosson, 1998; Schmitt, 2007), but the patterns of spatial genetic structuring, colonization, and microendemism in the Near East refugia have been obscured behind the sparse phylogeographic evidence. However, these areas have long been considered as refugia due to the presence of many Tertiary relict species. Nevertheless, the importance of phylogeographic patterns in the Near Eastern refugia is now becoming more well defined and appreciated. (Erichsen et al., 2018; Neiber & Hausdorf, 2015; Pokryszko, Cameron, Mumladze, & Tarkhnishvili, 2011; Tarkhnishvili, 2014; van Riemsdijk et al., 2017; Wielstra et al., 2010).

For many temperate species, hot spots of intraspecific genetic diversity have been identified within putative glacial refugia. Hot spots, areas where diversity has evolved (Petit et al., 2003), are supposed to be generated from two main evolutionary scenarios. Under the first scenario, the formation of hot spots for wide‐ranging temperate species would be a matter of long‐term survival of large, demographically stable populations in homogeneous refugia (Hewitt, 2000; Taberlet et al., 1998). Other fine‐scale phylogeographic evidence from populations within glacial refugia demonstrated that hot spots of genetic diversity may reflect the cycles of allopatric fragmentation within subrefugia which were followed by secondary contact and admixture of divergent lineages (Byrne, 2008; Canestrelli, Bisconti, Sacco, & Nascetti, 2014; Fijarczyk et al., 2011; Gomez & Lunt, 2007; Wielstra, ZieliŃski, & Babik, 2017). The second scenario suggests that refugia would represent evolutionary “melting pots” (Canestrelli, Aloise, Cecchetti, & Nascetti, 2010). Understanding the geographic distribution of hot spots of intraspecific diversity is not only important to enhance our knowledge in addressing fundamental questions in biogeography and speciation, but also is crucial for developing management plans for species conservation (Canestrelli et al., 2014 and citations therein).

**Figure 1:**
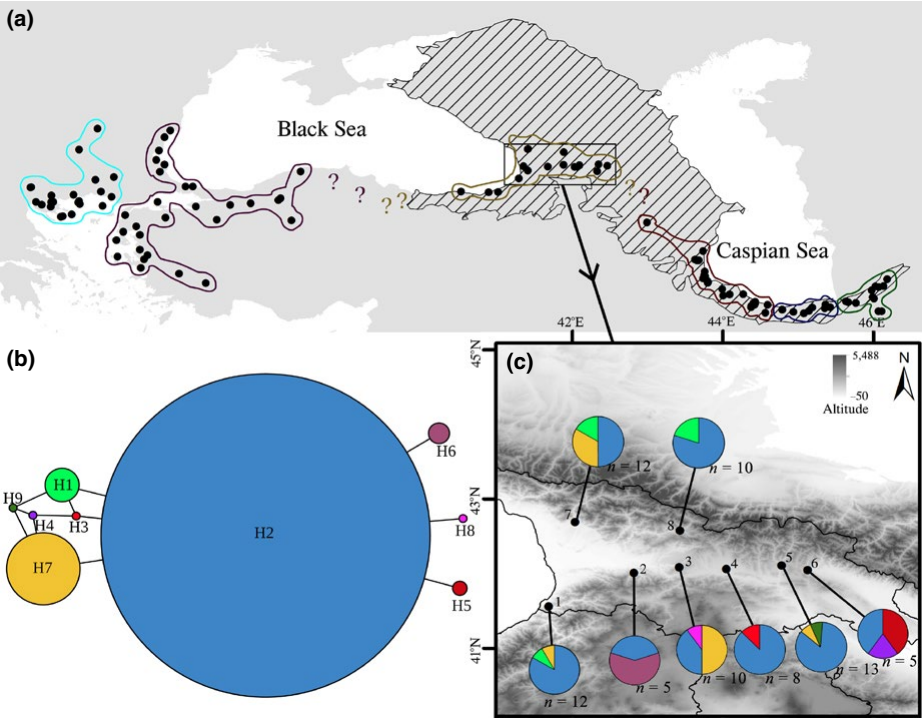
(a) The known distribution of *Potamon ibericum* species complex; the crosshatched region shows the Caucasus biodiversity hot spot and different groupings represent the geographic distribution of mtDNA lineages (see Supporting Information Figure S1 for more details); (b) Statistical parsimony network showing the frequency of each unique COI haplotype. The smallest colored circles represent single sample. (c) Sampling sites and geographic distribution of *P*. *ibericum*haplotypes in the western Caucasus. Numbers correspond to the localities as defined in Table 1. Pie charts on each locality represent the proportion of different haplotypes sampled by location

*Potamon ibericum* (Bieberstein, 1808) is a primary freshwater crab with a relatively wide range of distribution from river systems of the southern Caspian Sea to the Caucasus, through the river systems of the southern and western Black Sea, to the northern Aegean Sea in its western limit (Figure 1a; Brandis, Storch, & Turkay, 2000). Potamid crabs have limited dispersal abilities and seawater, large dry terrestrial landscapes and mountain ranges can act as their dispersal barrier (Parvizi, Naderloo, Keikhosravi, Solhjouy‐Fard, & Schubart, 2018; Shih, Zhou, Chen, Chien, & Ng, 2011; Yeo et al., 2008). They also lack pelagic larval dispersal, which intensifies their prolonged restriction to a freshwater system (Yeo et al., 2008) and consequently influences the spatial genetic pattern of their populations during colonization (Ibrahim, Nichols, & Hewitt, 1996). Previous studies on *P. ibericum* revealed the presence of at least seven separately evolving lineages within its total distribution (Jesse, Grudinski, Klaus, Streit, & Pfenninger, 2011; Parvizi et al., 2018). The eastern distribution of *P*. *ibericum* extends into two glacial refugia (Figure 1a): Hyrcania, located along the southern Caspian Sea, and Colchis, in the western part of the southern Caucasus (Tarkhnishvili, Gavashelishvili, & Mumladze, 2012). These areas altogether form the Caucasus biodiversity hot spot that is among the 25 most biologically rich hot spots worldwide, with considerable levels of endemism (Myers, Mittermeier, Mittermeier, Fonseca, & Kent, 2000). A recent phylogeographic study of *P*. *ibericum* inhabiting the drainages of Hyrcania elucidated three parapatrically distributed lineages, including western, central, and eastern Caspian lineages with substantial genetic diversity and strong population structure within each lineage (Parvizi et al., 2018). These findings showed that the Caspian Sea level fluctuations during glacial‐interglacial periods contributed to the cyclic expansion and contraction of populations from several independent local refugia in the southern Caspian Sea. Accordingly, the Hyrcanian region of the Caucasus biodiversity hot spot includes multiple Pleistocene refugia for the freshwater crab, *P. ibericum*. However, the importance of the Colchis region of the Caucasus biodiversity hot spot in shaping intraspecific genetic diversity of *P. ibericum* remains unclear. This study will provide evidence on whether the Colchis region harbored a single homogenous refugium or multiple independent local refugia for this taxon, or whether postglacial recolonization occurred from outside the Colchis. In addition, geographic structuring of genetic lineages in freshwater organisms, with special consideration of the vicinity of headwaters of the drainages in this area, is of importance and has remained unclear.

In the present study, we aim to understand the population genetic structure and demographic history of *P*. *ibericum* in the western parts of the Caucasus biodiversity hot spot, including the lowlands of Colchis and, to a smaller extent, the rivers that originate from highlands of the Greater and Lesser Caucasus. These areas include the refugial forests of the Caucasus as well as the sites where cold/arid condition and steppe vegetation were dominant during the LGM (Denk, Frotzler, & Davitashvili, 2001; Pokryszko et al., 2011). Due to the survival of Tertiary relict species, these areas are “traditionally” seen as important refugia. Nevertheless, more phylogeographic evidence will be essential to describe local refugia, postglacial dispersal dynamics, and levels of endemism in different taxa, especially the understudied freshwater crabs of these regions. By integrating genetic data from cytochrome oxidase subunit I (COI) sequences and paleoclimatic data from species distribution modeling, we explain the importance of the western Caucasus biodiversity hot spot in forming the intraspecific genetic diversity in *P*. *ibericum*.

## 2 MATERIALS AND METHODS

### 2.1 Sample collection and laboratory methods

Our studied area included eight localities across Georgia, covering the major geographic range of *Potamon ibericum* in this region (Figure 1a,c; Table 1). These localities included the drainage systems of both the Caspian Sea and the Black Sea. We used leg muscle tissue, preserved in absolute ethanol at −20°C, to extract genomic DNA, using a Puregene protocol (Gentra Systems, Minneapolis). Partial fragments of the cytochrome oxidase subunit I gene were amplified by polymerase chain reaction with primer pairs COL6 (Schubart, 2009) and COH10Po (Parvizi et al., 2018) following the procedures as described in Keikhosravi, Fratini, and Schubart (2015). The amplified products were sequenced by Macrogen (Seoul, South Korea). We read and manually corrected the resulting DNA sequences with Chromas v.2.6.2 (http://technelysium.com.au) and used the MUSCLE algorithm in MEGA 6 (Tamura, Stecher, Peterson, Filipski, & Kumar, 2013) to align the sequences and check amino acid translation for the presence of stop codons. Sequences of the different haplotypes have been deposited in the GenBank (http://www.ncbi.nlm.nih.gov) under accession numbers MK570620–28.

**Table 1:** Sampling details and genetic diversity indices for COI dataset (869 bp) of *Potamon ibericum*in the western Caucasus

No.	Local population	Drainage system	latitude	longitude	*N*	*h*	S	HD (*SD*)	*π*
1	Jochostskali	Black Sea	41.566267°	41.687067°	12	3‐H1‐H2‐H7	2	0.318 (0.164)	0.00038
2	Khanistskali	Black Sea	42.016017°	42.821033°	5	2‐H2‐H6	1	0.6 (0.175)	0.00069
3	Dzirula	Caspian Sea	42.092734°	43.422219°	10	3‐H2‐H7‐H8	2	0.644 (0.101)	0.00087
4	Dzama	Caspian Sea	42.011050°	43.955750°	8	2‐H2‐H3	1	0.25 (0.18)	0.00029
5	Aragvi	Caspian Sea	42.113717°	44.780317°	13	3‐H2‐H7‐H9	2	0.295 (0.156)	0.0005
6	Ilto	Caspian Sea	42.053250°	45.125350°	5	3‐ H2‐H4‐H5	3	0.8 (0.164)	0.00029
7	Jvari	Black Sea	42.697566°	42.039862°	12	3‐H1‐H2‐H7	2	0.667 (0.091)	0.00091
8	Rioni	Black Sea	42.584317°	43.430117°	10	2‐H1‐H2	1	0.356 (0.159)	0.00041
	Total				75	9	5	0.534 (0.064)	0.00074

Notes

Numbers correspond to the locality numbers as presented in Figure 1c.

*h*: number of haplotypes; Hd: haplotype diversity; *N*: number of samples; *S*: number of segregating sites; *π*: nucleotide diversity.

### 2.2 Genetic diversity and population structure

We evaluated diversity parameters including number of haplotypes (*h*), haplotype diversity (Hd), nucleotide diversity (*π*), and number of segregating sites (*S*) in DnaSP v.5.10 (Librado & Rozas, 2009) for each local population and for the complete dataset. Overall mean p‐distance was analyzed with MEGA v.6 (Tamura et al., 2013). We computed pairwise Φ_ST_ with 1,000 permutations in Arlequin v.3.5.2.2 (Excoffier & Lischer, 2010) to investigate population differentiation pattern among local populations (only populations with *n* ≥ 8 were included). Analysis of molecular variance (AMOVA) was performed to test for the genetic partitioning of the two main drainages (i.e., the Caspian Sea and the Black Sea drainage systems) by computing among drainage differences (Φ_CT_) and 1,000 permutations to test for significance in Arlequin v.3.5.2.2 (Excoffier & Lischer, 2010). In order to test if the haplotype distribution of *P. ibericum* in the Colchis and western Caucasus regions demonstrates a phylogeographic structure (*N*_ST_ > *G*_ST_; Pons & Petit, 1996), we calculated population differentiation (*G*_ST_) and the estimate of genetic differentiation for phylogenetically ordered alleles (*N*_ST_) with 10,000 permutations in SPADS 1.0 (Dellicour & Mardulyn, 2014).

We inferred relationships among *P. ibericum* COI haplotypes by a parsimony‐based haplotype network using TCS 1.21 with 95% cutoff (Clement, Posada, & Crandall, 2000). The haplotype network was edited and visualized in tcsBU (dos Santos, Cabezas, Tavares, Xavier, & Branco, 2016). In order to identify the relationship of the obtained haplotypes from this study with the previously recognized evolutionary lineages of *P. ibericum* species complex (Jesse et al., 2011; Parvizi et al., 2018), we retrieved COI sequences from GenBank (accession numbers MG729705–MG729772; HQ223156.1; HQ223158.1; HQ223162.1–67.1; HQ223201.1) and reconstructed a phylogenetic tree with Bayesian Inference (BI) by using MrBayes 3.2.2 (Ronquist et al., 2012). We used HKY+I as best‐fitting model of nucleotide substitution (previously selected with Jmodeltest 2.1.4, Darriba, Taboada, Doallo, & Posada, 2012) and conducted the BI analysis with four Markov Chain Monte Carlo (MCMC) simulations that were run for 5 000 000 generations and parameters sampled from each chain every 5000th generation. *Potamon kreation* and *Potamon setiger*were used as out‐groups (accession numbers HQ223177.1; HQ223198.1). The initial 25% of trees were discarded as burn‐in and the remaining trees were used to construct the Bayesian consensus tree. The resulting tree was viewed and edited in FigTree v.1.4.2 (Rambaut, 2014).

### 2.3 Demographic history

We tested for population expansion patterns in the Colchis and western Caucasus regions by estimating Fu's Fs (Fu, 1997) and Tajima's *D* (Tajima, 1989), and their significance through coalescent simulations with 1,000 permutations in DnaSP v.5.10 (Librado & Rozas, 2009). Fu's Fs statistic is based on the distribution of haplotypes, while Tajima's *D* is based on the frequency of mutations (Ramírez‐Soriano, Ramos‐Onsins, Rozas, Calafell, & Navarro, 2008). In addition, we calculated the distribution of pairwise differences (i.e., mismatch distribution; Rogers & Harpending, 1992) to trace population size change in DnaSP v.5.10 (Librado & Rozas, 2009). In order to measure the smoothness of the mismatch distribution, which discriminates between sudden population expansion or constant population size, we calculated Harpending's raggedness index (Harpending, 1994; Ramos‐Onsins & Rozas, 2002) and its significance level with 1,000 coalescent simulations in DnaSP v.5.10 (Librado & Rozas, 2009).

To assess how mtDNA effective population size changed through time, we analyzed historical demographics by using coalescent‐based Bayesian skyline plot (BSP) in BEAST 2.4.7 (Bouckaert et al., 2014). We selected F81+I as the best model of nucleotide substitution and considered a substitution rate of 2.33% per MY for COI (according to Schubart, Diesel, & Hedges, 1998). We set a strict clock model as prior and ran three independent MCMC analyses with 50 million generations, sampling every 5,000 steps, to verify the consistency of results. The results in log and tree files of independent runs were combined using LogCombiner v.2.4.7 (Rambaut & Drummond, 2017). The initial 25% of the samples were discarded as burn‐in. We tested the convergence of all parameters and produced BSP in Tracer 1.6 (Rambaut, Suchard, Xie, & Drummond, 2014).

### 2.4 Distribution modeling

To illustrate the suitable habitats and distribution modeling of *Potamon ibericum*, 13 localities were used. We retrieved localities' information from our field observations and from Brandis et al. (2000). Nine bioclimatic variables, including mean diurnal range (bio2), temperature annual range (bio7), mean temperature of warmest quarter (bio10), mean temperature of coldest quarter (bio11), precipitation seasonality (bio15), precipitation of wettest quarter (bio16), precipitation of driest quarter (bio17), precipitation of warmest and coldest quarter (bio18 and bio19), and altitude layer as a topology predictor, were applied to construct the current potential distribution of the species. The variables were selected based on the biology of the species, the previous species distribution modeling (SDM) on *P. ibericum* (Parvizi et al., 2018), and correlations among the 19 bioclimatic predictors and altitude layer. The correlation analysis was assessed by ENMTools version 1.4.3 (Warren, Glor, & Turelli, 2010) in order to avoid highly correlated and redundant variables.

To generate past distribution modeling of the species, the climatic data were extracted from the general circulation model (GCM) including the Last Glacial Maximum (LGM) scenario. The Community Climate System Model (CCSM4) and the earth system model (MIROC‐ESM) were used as the general atmospheric circulation models for this scenario. All the data were downloaded as raster format with 30 arc second for current layers and 2.5 min spatial resolution for the LGM. All the variables were extracted from WorldClim dataset (Hijmans, Cameron, Parra, Jones, & Jarvis, 2005).

Models were generated by Maxent version 3.3.3k as a general‐purpose machine learning method (Phillips, Anderson, & Schapire, 2006; Phillips, Dudík, & Schapire, 2004). Maxent is known to simulate species distribution using the minimum number of records and generate accurate species distribution models (Proosdij, Sosef, Wieringa, & Raes, 2016). In all models' settings, 10 cross‐validate replicated run types were used. Also, we set 10,000 randomly background points as pseudo‐absence in the entire studied area, 1,000 maximum iterations with a 10^−5^ convergence threshold and regularization multiplier of 1. Logistic output format was set to describe a continuous probability of presence which ranges between 0 and 1 (Phillips & Dudík, 2008). The average maps of each analysis were used as final outputs to identify the potential distribution of *P. ibericum*. The final maps in ASCII format were visualized and processed using ArcGIS version 10.2^®^ (ESRI, 2013).

To evaluate the predicted models, a threshold‐independent receiver operating characteristic (ROC) analysis (Elith et al., 2006) was used. Considering the ranges of the area under the curve (AUC) derived from ROC plot, AUC is valued from 0 to 1. A model with an AUC value higher than 0.75 (Pearce & Ferrier, 2000) indicated acceptable and robust model. The importance of each predictor was explored by Jackknife analysis of regularized training gain.

The lowest presence threshold (LPT) (Pearson, Raxworthy, Nakamura, & Townsend Peterson, 2007) as a threshold‐dependent test, was applied to represent the locations that were at least as suitable as those where the species has been recorded (Hernandez, Graham, Master, & Albert, 2006; Pearson et al., 2007).

## 3 RESULTS

### 3.1 Phylogeographic pattern and demographic history

Overall, moderate haplotype diversity (0.534, *SD* = 0.064), low mean nucleotide diversity (0.00074), and shallow evolutionary divergence (mean uncorrected *p*‐distance = 0.00007) were observed in *Potamon ibericum* within the studied area. We found only nine unique haplotypes among 75 COI sequences (Figure 1b and Table 1). There is a clear evidence of gene flow between the studied rivers as revealed by pairwise Φ_ST_ values (Table 2). Also, hierarchical AMOVA did not show significant genetic partitioning between the two main drainages of the region (COI among‐drainages variance = –2.98%, *df* = 1, *F*_CT_ = –0.029, *p* = 0.5). The lack of phylogeographic structure was approved from phylogeographic structure analysis (*N*_ST_ = 0.1728, *G*_ST_ = 0.1785, *p* = 0.48).

**Table 2:** Pairwise Φ_ST_ values representing COI genetic structuring between the studied localities (*n* ≥ 8) of *Potamon ibericum* in the western Caucasus

Locality	Rioni	Dzama	Jvari	Dzirula	Aragvi
Dzama	–0.013 (ns)	0			
Jvari	0.088 (ns)	0.112 (ns)	0		
Dzirula	0.305*	0.287*	–0.006 (ns)	0	
Aragvi	0.008 (ns)	–0.004 (ns)	–0.013 (ns)	0.129 (ns)	0
Jochostskali	–0.032 (ns)	–0.042 (ns)	0.038 (ns)	0.214*	–0.072 (ns)

* Significant values (*p* > 0.05) from 1,000 permutations, ns indicates nonsignificant values and lack of genetic structuring.

The phylogeographic reconstruction of *P. ibericum* revealed that the haplotypes of Colchis and western Caucasus are clustered with the previously recognized haplotypes of *P. ibericum tauricum*from the localities Hemsin and Goreme in northeast Turkey (Supporting Information Figure S1). These haplotypes altogether correspond to the “Eastern Black Sea lineage” of the *P. ibericum* species complex (see Parvizi et al., 2018).

**Figure 2:**
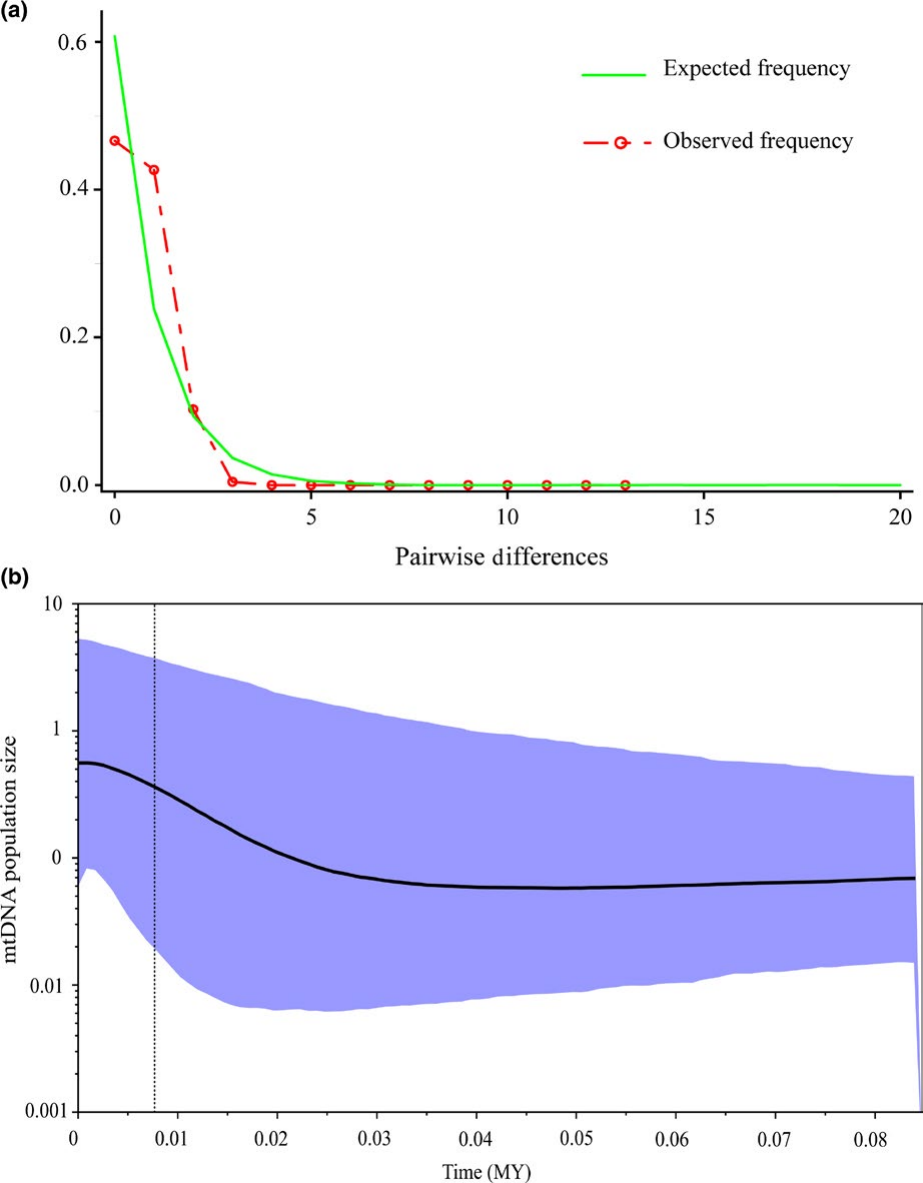
(a) Unimodal distribution of the numbers of pairwise COI differences among *Potamon ibericum* individuals in the western Caucasus. (b) Bayesian skyline plot shows population size change over time. Maximum time in the *X*‐axis is the root height mean, dotted vertical line represents lower 95% highest posterior density (HPD). Horizontal line represents the median parameter estimate with the 95% HPD interval

The evidence of population expansion was provided by the demographic analyses. Negative values of Fu's Fs (–5.6, *p* = 0.003) and Tajima's D (–1.1, *p* = 0.1) tests support an excessive number of rare haplotypes as a result of a recent population expansion (Fu and Li, 1993; Fu, 1997). Nevertheless, the nonsignificant Tajima's *D* restricted our inference about demographic expansion based on this analysis. The distribution of pairwise haplotype differences was skewed to the left, which supports a sudden population expansion hypothesis (Figure 2a). Additionally, positive and statistically nonsignificant Harpending's raggedness index failed to reject sudden demographic expansion in the region (*r* = 0.1, *p* = 0.14). A Bayesian skyline plot showed an increase in the mtDNA effective population size toward the present with a marked expansion which began at about 25,000 years BP (Figure 2b).

### 3.2 Distribution modeling

The generated models based on 13 presence records of the western Caucasus performed well and presented good models with a Maxent‐generated AUC evaluation. The potential distribution models of *P. ibericum* showed good AUC test value, with 0.92 ± 0.01 and 0.90 ± 0.01 for training and test data, respectively. Furthermore, the binomial omission test with the lowest presence threshold was statistically significant and the test omission rates did not exceed 5%.

According to the Jackknife analysis of regularized training gain (Supporting Information Figure S2), when used in isolation, precipitation of driest quarter (bio17) was the strongest predictor. The next important variable is precipitation of coldest quarter (bio19). Following these two variables, the species was also influenced by mean temperature of coldest quarter (bio11), precipitation of wettest quarter (bio16), precipitation seasonality (bio15), and altitude.

**Figure 3:**
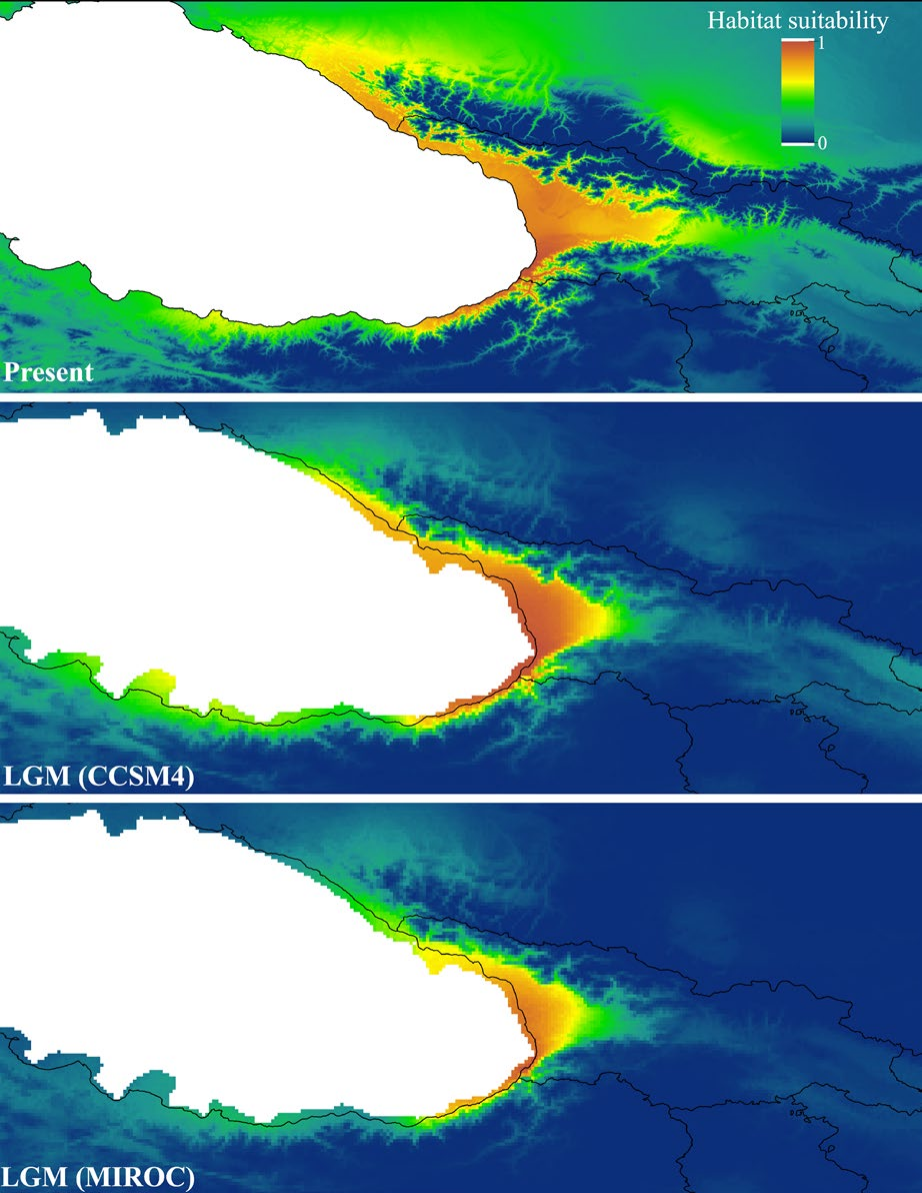
Species distribution modeling predictions of *Potamon ibericum* of the western Caucasus, representing distribution models of present and the Last Glacial Maximum (LGM) based on Community Climate System Model (CCSM4) and Model for Interdisciplinary Research on Climate (MIROC)

Under current bioclimatic conditions (Figure 3), the model predicts highly suitable areas along the Black Sea coasts and the drainages of the Greater and Lesser Caucasus. This fits well with the current distribution of *P. ibericum* in these regions. The projection of the model over the LGM layers (Figure 3) represented high habitat suitability along the Black Sea coasts and Colchis region irrespective of the global circulation models.

## 4 DISCUSSION

### 4.1 Population structure, demographic history and glacial refugia

There is relatively little known about the intraspecific diversity and population demography of *Potamon ibericum* in the western Caucasus. The last comprehensive study on the zoogeography and taxonomy of potamid crabs (Brandis et al., 2000) provided only one record of *P. ibericum* in Natanebi, Georgia, and two records from an undetermined Caucasian country. Accordingly, the present study is the first comprehensive phylogeographic study of a potamid crab in this region.

Although we found private haplotypes (H3, H4, H8, H9) in four localities, including Dzirula, Dzama, Aragvi, and Ilto, the overall lack of geographic and population genetic structure was confirmed in the studied area. Most of the haplotypes are present in more than one population (Figure 1c), which implies extensive gene flow among populations. Freshwater crabs have limited dispersal abilities, are unable to surmount terrestrial, marine and mountainous barriers, and are highly philopatric and restricted to a freshwater system even for their lifetime (Daniels, Gouws, & Crandall, 2006; Poettinger and Schubart, 2014; Shih et al., 2011; Yeo et al., 2008). However, populations from nearby drainages can disperse over short land bridges during periods of heavy rainfall which will result in an invariant population genetic structure within a drainage system, as it was previously reported for freshwater crabs of the family Potamonautidae (Daniels, Stewart, & Cook, 2002; Daniels, Stewart, & Gibbons, 1998,1999), Pseudothelphusidae (Schubart, Rivera, Crandall, & Santl, 2011) and Potamidae (Keikhosravi et al., 2015). Some studies have also suggested that freshwater crabs can potentially disperse over short terrestrial barriers under high humidity conditions (Gherardi, Tarducci, & Vannini, 1988; Phiri & Daniels, 2014). The considerable shared polymorphism of *P. ibericum*among the rivers of Colchis and western Caucasus confirms the possibility of gene flow within a drainage system. The nonsignificant genetic partition between the two main drainages, the Caspian Sea Basin and the Black Sea Basin, determined by hierarchical AMOVA. AMOVA results also strongly support that different drainages cannot act as a barrier to dispersal if environmental variables, such as precipitation, temperature and topographic conditions, are in the favor of unconstrained gene flow in *P. ibericum* of the western Caucasus (Supporting Information Figure S2; see SDM results). Such inference can also be drawn from the continuity of suitable habitats for *P. ibericum* in the studied area under the current climate scenario (Figure 3). It is worth noting that when all studied individual crabs share a quite recent common ancestry (inferred from low pairwise Φ_ST_ values and the star‐like shape of the haplotype network) and when the evidence of recent and rapid demographic expansion in the region is pronounced, it would not be implausible to observe genetic homogeneity across a relatively small and homogeneous landscape like the Colchis and western Caucasus. A thorough genetic sampling would unravel true haplotype diversity across this region.

We found congruent evidence of a recent and rapid demographic expansion from different demographic analyses, including negative and significant Fu's Fs, unimodal mismatch distribution (Figure 2a), and positive and nonsignificant Harpending's raggedness index. The analyses of population genetic indices, including moderate haplotype diversity, low nucleotide diversity, and shallow genetic divergence also imply a recent and rapid population expansion. The coalescent‐based BSP shows relatively slight increase in female effective population size during the LGM, which is followed by a rapid postglacial expansion (Figure 2b). Moreover, SDM, in line with molecular findings, shows the maintenance of highly suitable habitats for *P. ibericum* along the eastern and southeastern coasts of the Black Sea and in Colchis (Figure 3). The area of predicted species presence during the LGM varies between circulation models; nevertheless, it has been suggested that for temperate forest species of the Caucasus, the MIROC climatic simulation provides a more realistic pattern of the LGM climate rather than the CCSM simulation (Tarkhnishvili et al., 2012). Due to their complex geography and peculiar topography, the eastern coasts of the Black Sea are well‐known refugia for many plant and animal taxa (Denk et al., 2001; Kikvidze & Ohsawa, 2001, Pokryszko et al., 2011; Sochor & Trávníček, 2016; Tarasov et al., 2000; Wielstra et al., 2013). Our results altogether strongly support that Colchis and western Caucasus served as a glacial refugium for the freshwater crab, *P. ibericum*. Although some studies imply multiple, independent glacial forest refugia in these areas (Neiber & Hausdorf, 2015; Tarkhnishvili, Thorpe, & Arntzen, 2000), our results suggest a homogeneous and large refugium along the eastern Black Sea coast.

The major forest refugium (MFR) is an area between the western Lesser Caucasus and northeast Turkey which, based on paleontological data, harbored a homogeneous refugium during the LGM (see Mumladze, Tarkhnishvili, & Murtskhvaladze, 2013). Phylogeographic studies on the Caucasian Salamander, *Mertensiella caucasica* (see Tarkhnishvili et al., 2000), and the large endemic Caucasian snail, *Helix goderdziana* (see Mumladze et al., 2013), have shown that the ancestors of these taxa survived in the MFR. Based on the phylogenetic reconstruction of the *P. ibericum* species complex, the Colchis and western Caucasus haplotypes clustered together with individuals from northeast Turkey, forming the Eastern Black Sea lineage, without revealing any phylogeographic break in this region (Figure 1a and Supporting Information Figure S1). Additionally, suitable habitats for *P. ibericum*were uninterruptedly available during the LGM in these areas (Figure 3). Therefore, our results imply that the ancestral Eastern Black Sea lineage of *P. ibericum* survived in a homogeneous refugium that extended from Colchis to the MFR. These areas are among the few regions in the western Palearctic where mixed broadleaf forests and warm and humid condition were dominant during the LGM as a consequence of warm and humid winds across the Black Sea, while northern, southern, and eastern areas were characterized by extreme cold or arid condition with steppe vegetation (Denk et al., 2001; Pokryszko et al., 2011). Here we present evidence concerning the notion that the presence of a rather homogenous and large refugium in the northeast Turkey and Colchis can be inferred from those species “confined to,” but not “restricted within” these areas (Pokryszko et al., 2011).

### 4.2 Intraspecific genetic diversity of *P. ibericum* in the Caucasus biodiversity hot spot

The Caucasus biodiversity hot spot connects two distinct zoogeographic regions of the western Palearctic: the Euro‐Siberian and Irano‐Turanian (Zazanashvili, Sanadiradze, Bukhnikashvili, Kandaurov, & Tarkhnishvili, 2004). This region encompasses the mountain ranges of the Greater and Lesser Caucasus and the Alborz mountains in the southern Caspian Sea. Generally, biodiversity is often higher in mountainous regions because mountains provide topographic complexities and ecological gradient which can affect diversification and endemism (Hoorn, Mosbrugger, Mulch, & Antonelli, 2013; Noroozi et al., 2018). Accordingly, species diversity and endemism are remarkably high in the Caucasus biodiversity hot spot. In addition, the presence of two important western Palearctic glacial forest refugia, Colchis and Hyrcania, has contributed to the evolution of divergent lineages and considerable levels of intraspecific diversity.

The interaction between climatic fluctuations during the Pleistocene and landscape features of the Caucasus biodiversity hot spot have initiated the microevolutionary processes that shaped the current phylogeographic structure of *P. ibericum* in this region. Following postglacial expansions, the northern coasts of the Black Sea and the drainages of the central Caucasus were colonized from Colchis and the MFR, while the populations within a Hyrcania refugium colonized westward through the drainages of Azerbaijan. Although, there is still lack of information on the genetic pattern and the LGM geographic ranges of *P. ibericum*in the Azerbaijan region, our single genetic sample from Karabakh, Azerbaijan (voucher specimen SMF 2730, unpublished data) suggests a Hyrcanian origin of populations in this region. Our findings together with previous study on *P. ibericum*suggest a pattern of “refugia‐within‐refugia” in the Hyrcanian regions of the Caucasus biodiversity hot spot, in contrast to a large homogenous refugium in the Colchis region. In order to clarify the phylogeographic breaks and the spatial distribution of evolutionary lineages of potamids in the Caucasus biodiversity hot spot, we recommend that future studies pursue a more thorough investigation of the drainages of Azerbaijan and incorporate variable nuclear markers.

## ETHICAL APPROVAL

All applicable international, national, and/or institutional guidelines for the care and use of animals were followed.

## CONFLICT OF INTEREST

The authors declare that they have no conflict of interest.

## AUTHOR CONTRIBUTIONS

E.P., R.N., A.K., and C.D.S. conceived the study; R.N. and A.K. carried out the fieldwork; S.S.F. performed the SDM analysis; F.S. performed genetic laboratory work. E.P. performed genetic analysis and drafted the manuscript. All authors corrected and approved the final version of the manuscript.

## Data Availability

All relevant data are within the paper and its Supporting Information files. The DNA Sequence data has been submitted to GenBank under the accession number MK570620‐28.
