# Supplementary figures and images for: Phylogeography of Potamon ibericum (Brachyura: Potamidae) identifies Quaternary glacial refugia within the Caucasus biodiversity hot spot
Source: Ecol Evol. 2019 Mar 26;9(8):4749–59. doi: 10.1002/ece3.5078 (PMC6476761; doi:10.1002/ece3.5078)

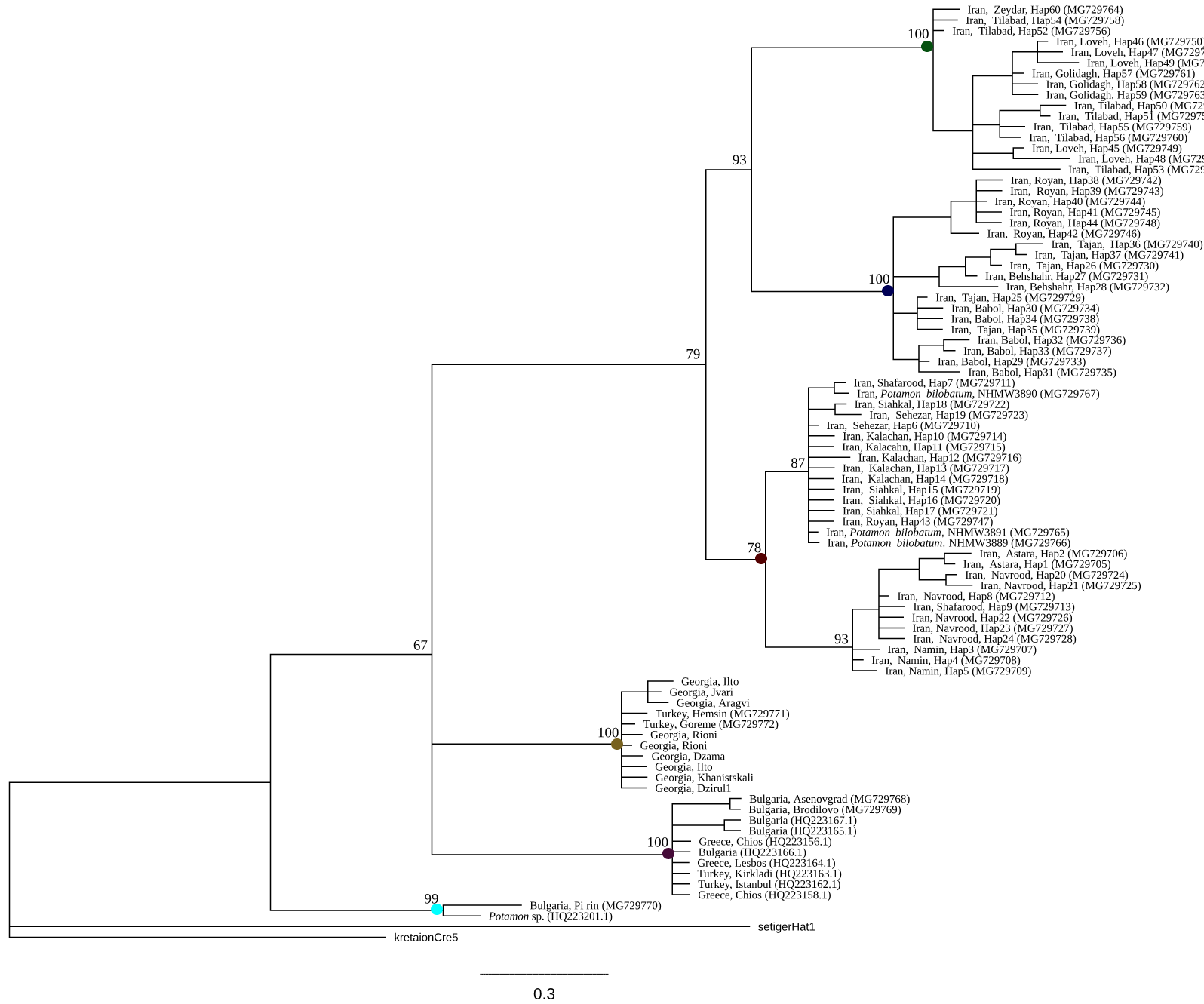

Supplement: Supplementary file 1 [file ECE3-9-4749-s001.tiff]

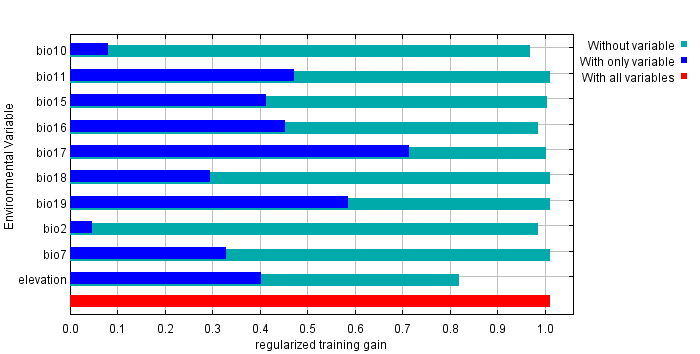

Supplement: Supplementary file 2 [file ECE3-9-4749-s002.png]
