# Supplementary material for: Phylogeography of Potamon ibericum (Brachyura: Potamidae) identifies Quaternary glacial refugia within the Caucasus biodiversity hot spot
Source: Ecol Evol. 2019 Mar 26;9(8):4749–59. doi: 10.1002/ece3.5078 (PMC6476761; doi:10.1002/ece3.5078)
Supplement: Supplementary file 3 [file ECE3-9-4749-s003.docx]

**Supplementary figure legends:**

**Fig. S1** phylogenetic reconstruction based on COI illustrating separately evolving lineages within *Potamon ibericum* species complex. The obtained unique haplotypes of the Colchis and western Caucasus are clustered with the previously published haplotypes of Hemsin and Goreme localities of northeast Turkey. These haplotypes altogether represent the Eastern Black Sea lineage.

**Fig. S2** Jackknife analysis of regularized training gain for *Potamon ibericum* of the Colchis and western Caucasus.
